# Bibliometric, methodological and reporting characteristics of systematic reviews with explicit AI disclosure statements: an exploratory meta-research study

**DOI:** 10.1186/s12874-026-02796-2

**Published:** 2026-02-11

**Authors:** Anastasios Bastounis, Lukasz Lagojda, William Sheppard, George Daly, Edith Poku, Andrew Booth

**Affiliations:** 1 Division of Population Health, School of Medicine and Population Health, Sheffield Centre for Health and Related Research (SCHARR), University of Sheffield, Regent Court, 30 Regent Street, Sheffield, S1 4DA UK; 2 Warwickshire Institute for the Study of Diabetes, Endocrinology and Metabolism (WISDEM), University Hospitals Coventry & Warwickshire NHS Trust, CV2 2DX Coventry, UK; 3 School of Psychology, Faculty of Medicine and Health, University of Leeds, University Road, Leeds, LS2 9JT UK

**Keywords:** Meta-research, Systematic reviews, Methods, Reporting, LLMs, ChatGPT

## Abstract

**Introduction:**

The exponential increase in systematic reviews (SRs), accelerated by LLM-based generative AI and non-LLM automation tools, risks redundancy, overlap, and research waste. However, there is limited empirical evidence on how SRs that disclose AI use apply and report these tools in practice, including the extent of transparency and validation.

**Aim:**

To assess the methodological and reporting features of SRs that explicitly acknowledge LLM-based and non-LLM automation tools’ use in a dedicated statement, and to examine how these features relate to bibliometric characteristics of these SRs.

**Methods:**

An exploratory, cross-sectional, meta-research study with individual SRs as the unit of analysis. A random sample was drawn from a purposively defined stratum, comprising only SRs with designated AI statements. Screening was conducted by a single researcher; data extraction was performed by one researcher and independently verified by four others. Descriptive analyses were supplemented by Wilcoxon rank-sum tests, Spearman’s *ρ*, and *χ*² tests.

**Results:**

We included 188 SRs; 75% reported using LLMs, and in 92% of studies LLM-based and non-LLM automation tools were used for manuscript writing. Reviews with designated AI statements were predominantly published in Elsevier or Elsevier-supported journals (70.2%). Only 42% referenced a pre-registered protocol; the median time from protocol registration to first journal submission was 267 days. Reviews with more included studies were published in higher-impact journals (*ρ* = 0.34, *p* < 0.0001), as were reviews led by authors affiliated with high-income countries (*W* = 1931.5, *p* < 0.0001). Reviews with more authors were more likely to have a pre-registered protocol (*χ*² = 20.54, *p* < 0.0001), and pre-registered reviews more often adhered to a reporting checklist (*χ*² = 8.93, *p* = 0.0027).

**Conclusions:**

LLM-based and non-LLM automation tools were used predominantly for writing. Sharing of prompts and human-validation procedures was insufficient, and many reviews exhibited methodological and reporting weaknesses. Clearer guidance is needed to support transparent, rigorous use of LLM-based and non-LLM automation tools in SRs.

**Supplementary Information:**

The online version contains supplementary material available at 10.1186/s12874-026-02796-2.

## Highlights

Early experience with LLM-based and non-LLM automation tools, as reflected in dedicated AI statement sections, indicates that in most cases these tools were reportedly used solely for proofreading and writing assistance, predominantly via ChatGPT.

These SRs were more likely to be published by Elsevier or Elsevier-supported journals.

Reviews led by authors affiliated with institutions in high-income countries and including more studies tended to be published in higher-impact journals.

Reviews with more authors were more likely to have a pre-registered protocol, and those with a pre-registered protocol were more likely to have adhered to a reporting checklist.

Approximately 40% of the included SRs that have conformed to a checklist, either cited outdated versions, misused them or provided insufficient documentation for adherence to a reporting checklist.

**Supplementary Information:**

The online version contains supplementary material available at 10.1186/s12874-026-02796-2.

## Introduction

Systematic reviews (SRs) offer a research methodology for collating, synthesising, and critically appraising data drawn from collections of individual studies. They are particularly well-suited for identifying research gaps, informing theory development, estimating the diagnostic accuracy of medical tests, and assessing the effectiveness of interventions and medical devices [1]. Renowned for aiming to be transparent, reproducible, and easily updated, SRs have shaped how research evidence is translated into healthcare and clinical practice [2]. Due to their potential methodological rigour, SRs are often labelled the “gold standard” for informing evidence-based practice [3]; however, in practice, the quality of published SRs can vary substantially.

The past two decades have witnessed an exponential increase in publication of SRs in peer-reviewed journals [4]. Future acceleration will be arguably attributable, at least in part, to the growing uptake of AI-assisted technologies (e.g., automation tools) as they streamline the conduct and production of SRs [5]. Further evidence for the impact that AI can have in streamlining the conduct and expediting the production of SRs comes with the emergence of Large Language Models-based generative AI (LLMs hereafter), which can accelerate the review process from inception to dissemination. LLMs are a type of deep-learning model (a subset of machine learning), and machine learning is a subset of AI. Despite LLMs tremendous potential, the significant methodological and ethical challenges that can impede scientific reasoning and undermine research integrity [6, 7], naturally extend to cover the conduct and publication of SRs. Suboptimal implementation of the methods underpinning SR production, failure to adequately investigate sources of heterogeneity, and non-adherence to reporting checklists can all influence the synthesis of findings and weaken the scientific validity of SR conclusions [8–10]. Although efforts to standardise the conduct and reporting of evidence syntheses that incorporate AI-assisted tools are being made [11], there is currently no consensus addressing the use of LLMs in SRs.

Given the rapid proliferation of SRs and the availability of accessible LLMs, we can project an even greater increase in SR production over the near future. Potential repercussions may be encountered in connection with the research integrity of SRs, overlap between reviews, and the duplication and waste of research person-power. In light of recent attempts to provide concrete guidance regarding the use of AI within SRs, there is a need to investigate the methodological, reporting, and bibliometric characteristics of SRs that have acknowledged the use of AI in their conduct so far. As a starting point, the evaluation of key characteristics of the stratum of SRs reported in this paper, and how these characteristics are associated with publication patterns, will inform the development of specialised guidance around the use of AI within SRs and evidence syntheses in general.

An exploratory, cross-sectional, meta-research study design was identified as appropriate. Meta-research has been found to be well-suited to evaluate and critically appraise SRs and to investigate how and to what extent methodological and reporting characteristics affect the observed effects [12–14]. The aim of this study is to investigate the methodological and reporting characteristics of those SRs that have explicitly reported, in a designated statement, the use of LLM-based and non-LLM automation tools at any stage of the SR process. This study addresses three objectives: (i) to map the use of LLM-based and non-LLM automation tools across review stages (ii) to describe the methodological and reporting characteristics of these SRs; and (iii) to examine how these characteristics are associated with the bibliometric characteristics of these SRs in peer-reviewed journals.

## Methods

### Study design

This exploratory meta-research study employs a cross-sectional design with individual systematic reviews as the unit of analysis. The protocol of this meta-research study was prospectively registered with Open Science Framework (OSF) (https://osf.io/uy2pc*)* [15]. In the absence of a dedicated reporting checklist for meta-research [16] and given the specific idiosyncrasies of this study (e.g., identify and analyse multiple studies), the reporting of this exploratory meta-research study was guided by the adapted version of the PRISMA 2020 checklist for reporting systematic reviews [17].

### Eligibility criteria

Eligible studies for inclusion were SRs that have explicitly disclosed in a designated ‘*Declaration of Generative AI and AI-Assisted Technologies*’- type statement, the use of LLM-based and/or non-LLM automation tools at any stage of the SR process. The full list of eligibility criteria is presented in the supplementary material and the designated OSF webpage of this study.

### Information sources and search strategy

Reviews that include a designated AI-use statement are difficult to retrieve via conventional bibliographic database searching because these statements are typically located in end-matter (e.g., publisher-required disclosure sections) and are not consistently indexed in standard metadata fields. In pilot scoping searches prior the protocol upload to OSF, conventional database searches using generic AI/LLM-related keywords yielded a very large volume of irrelevant records (e.g., articles about AI) while still risking omission of eligible SRs in which AI use was disclosed only in end statements. We therefore used a targeted Google Scholar approach (via Publish or Perish^1^) to identify SRs containing designated AI statements, using highly specific query combinations designed to maximise precision: systematic review [title] AND “AI Statement:”; systematic review AND “Use of Generative AI”; systematic review AND “Declaration of Generative AI”; systematic review AND “Declaration of Generative Artificial Intelligence”; and systematic review AND “Use of Generative Artificial Intelligence”. The searches in Google Scholar were conducted on 14th May 2025. The number of records retrieved for each combination are provided in the supplementary materials.

### Data collection process, data items and variables

Bibliographic references for selected SRs were imported into Excel and subsequently deduplicated. One researcher (ABa) conducted a two-phased study selection using pre-specified eligibility criteria: initial screening of all titles and abstracts, followed by full-text assessment of potentially eligible studies. A data extraction form was developed and was pilot-tested using two SRs with AI-use designated statements among three researchers (ABa, LL, EP). This pilot exercise permitted us to decide on the most appropriate categories to be included in relation to the AI use, the AI/LLM tools used as well as the breadth of validation and human involvement, ensuring that all relevant categories would be captured. One researcher conducted the extraction across all included SRs (ABa). Four additional, “unblinded” to study characteristics researchers (LL, WS, GD, EP), independently verified the extracted data with approximately equal distribution of SRs per researcher (*n* ≈ 47 SRs per researcher). Any discrepancies in data extraction identified through the verification process were resolved through 1:1 discussion between the extractor (ABa) and the rest of the researchers. All the data extraction sheets from the lead extractor and the researchers who conducted the verifications were uploaded to the dedicated OSF webpage prior to submission of the manuscript.

Data on the following categories were extracted: studies’ metadata, country of the institutional affiliation of the first author, journal where the study was published, publisher of the journal, journal’s impact factor (IF), discipline where the SR topic lies, size of the author team (i.e., small team: ≤3), ethnicity of the author team (i.e., National or International as denoted by the presence of at least two authors with different main country affiliations), presence of a funding statement, presence of conflict of interest statements, presence of a data availability statement, presence of a pre-registered protocol, time between protocol registration and first submission to the journal, adherence to reporting checklist, evidence supporting adherence to the checklist, number of individual studies included in the SRs, evidence that risk of bias assessment was conducted independently in duplicate, evidence that title/abstract screening was conducted independently in duplicate, evidence that full-text screening was conducted independently in duplicate, whether meta-analysis and certainty of evidence were included, type of AI tool, name of AI tool, review stage where the AI tool was used, purpose of AI use, description of AI tool, AI prompts disclosure/prompt sharing, validation of AI content, and human involvement. The completed data extraction sheet is included in the supplementary material.

### Statistical methods

Descriptive statistics were reported for the methodological, reporting, and bibliometric characteristics of the included SRs. Medians and interquartile range (IQR) were calculated for continuous variables and counts with percentages were reported for binary variables. Counts and frequencies were reported for AI-related variables. A total of eight bivariate statistical tests (i.e., Wilcoxon rank-sum tests, Spearman’s rank correlation, and chi-squared tests) were conducted to assess associations among the variables of interest. We hypothesised that certain reporting and methodological characteristics would be associated with publication in higher-impact journals. We expected that SRs with a pre-registered protocol, lead authors affiliated with institutions in high-income countries, large author teams, international collaborative author teams, a higher number of included studies, and adherence to reporting checklists would be associated with higher journal impact factors. Additionally, we hypothesised that SRs with a pre-registered protocol would be more likely to (i) adhere to a reporting checklist and (ii) be authored by large teams. In line with our protocol and to account for multiple testing (eight statistical tests), the statistical significance threshold was adjusted to *p* ≤ 0.00625 after applying the Bonferroni correction. All analyses were conducted using R version 4.3.1 [18]. The following R packages were used to create the graphs and conduct the analysis: “ggplot2” [19], “plotly” [20], “ggalluvial” [21], “dplyr” [22].

## Results

The verification process revealed that approximately 24% of the included SRs (*n* = 45) required a discussion to reach consensus on one or more variables, with absolute agreement between the extractor and the verifying researchers in 76% of studies. Overall, 12 of the 31 extracted variables were affected by disagreements, most frequently for: (i) the time lag between protocol registration and first submission to the journal; (ii) whether PRISMA checklist use was sufficiently supported; and (iii) whether independent screening (title/abstract and full text) and data extraction were conducted by two reviewers. Disagreements between the extractor and verifiers in AI-use related variables were observed in only one SR. All the extraction sheets from both the extractor and the verifying researchers were uploaded to the designated OSF webpage prior to the submission of the manuscript.

### Descriptive statistics on the use of LLM-based and non-LLM automation tools

**Fig. 1:**
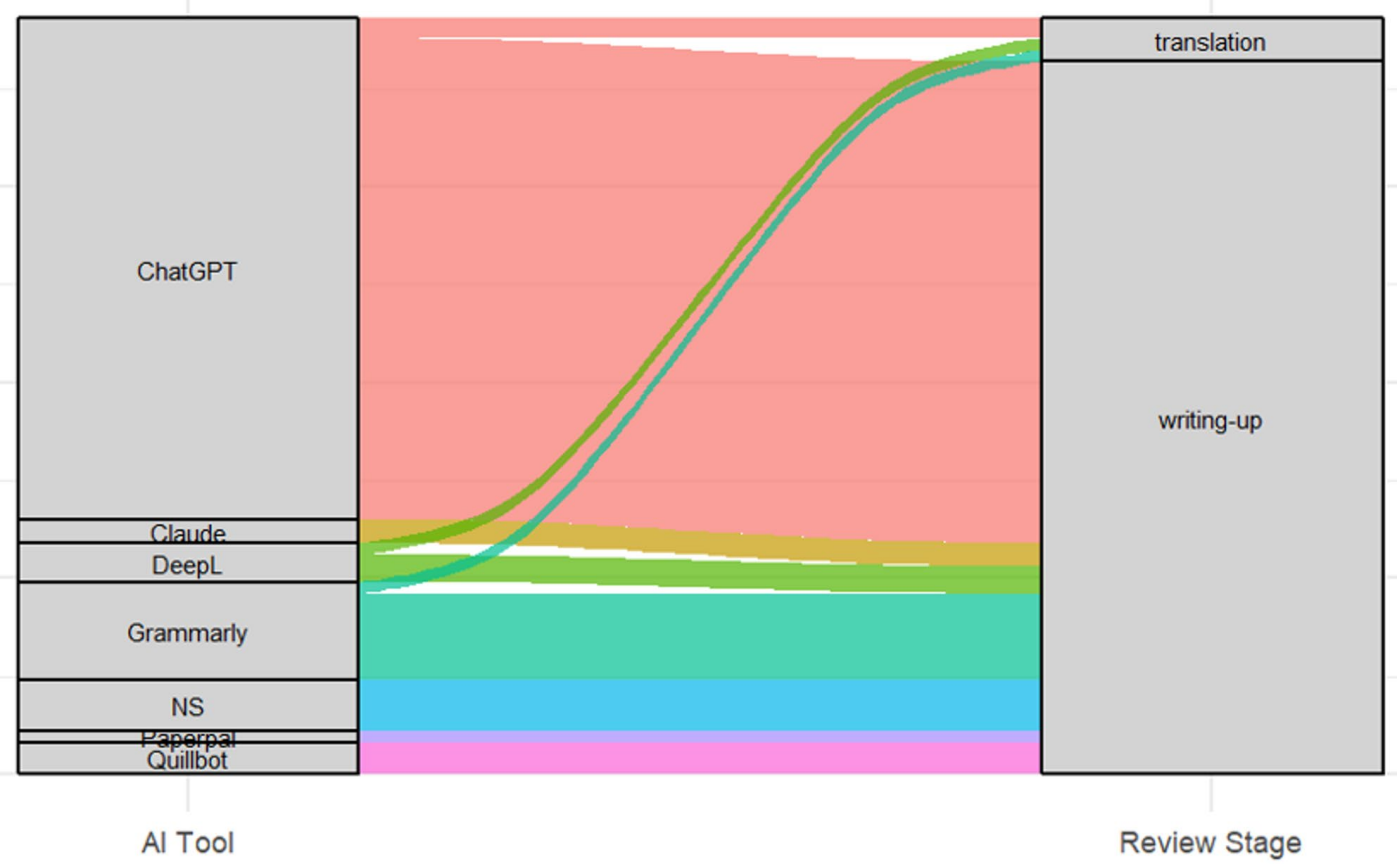
*Alluvial diagram showing the distribution of the most-frequently used AI-based tools (LLM and machine-learning based AI tools) used in the selected SRs and their application across review stages. *Note.* NS: Not stated (name of the tool). *White gaps between the strata blocks indicate other tools that have been rarely used. In total, 23 other machine-learning-based tools (not depicted in the diagram) were used across 26 SRs mainly for writing-up purposes (including proof-reading) and translation

Overall, 188 SRs explicitly reported use of LLM-based and non-LLM automation tools, and were included in this meta-research study. In total, 75% (*n* = 141) reported the use of LLMs (not AI-assisted tools) in a designated statement, followed by 24 SRs (12.8%) that used AI-assisted tools (not LLMs), and 21 SRs (11.2%) that used both AI-assisted tools and LLMs. Overall, 70.2% (*n* = 132) of the identified SRs were published by Elsevier or Elsevier-supported journals, followed by Springer (*n* = 8, 4.3%) and Taylor & Francis journals (*n* = 7, 3.7%). In 92% of the selected records (*n* = 173), LLM-based and/or non-LLM automation tools were reportedly used solely for writing-up purposes (e.g., proofreading, refining text or enhancing language flow). In 69% (*n* = 130), SRs reportedly used different versions of ChatGPT (Fig. 1; Appendix III).

Only two of the 188 selected SRs (1%) provided a generic description of the function of the LLM that was used and a further two SRs reported the prompts used in the LLMs. In three out of these four cases, authors had used ChatGPT for writing-up purposes only. In one out of four SRs, authors used ChatGPT for writing-up purposes and for generating statistical code. There was a uniformity in the statements provided regarding the validation of the AI content (this material is freely available via the designated OSF webpage for this study and was uploaded prior to submission of the manuscript).

### Descriptive statistics on the methodological, reporting, and bibliometric characteristics

In the SRs explicitly acknowledging the use of LLM-based and/or non-LLM automation tools, most authors had affiliations with institutions in high-income countries (*n* = 105, 55.9%), however most SRs were located in non-Western countries (China: 15, 7.98%; Iran: 23, 12.23%). The median time for a SR with an AI-designated statement to proceed from protocol registration to first submission to the journal was 267 days (IQR: 424), while the median impact factor of the journal where these SRs were published was 3.6 (IQR: 3.05). Most of these SRs addressed topics that fall broadly within medical disciplines (*n* = 124, 65.96%). Most of the SRs were authored by large author teams, authored by national teams, and included funding, conflict of interest, and data availability statements (Table 1). Most of the reviews did not report a pre-registered protocol (*n* = 109, 58%), while most SRs claimed to adhere to a reporting checklist (*n* = 154/188, 81.9%). In most of the cases (*n* = 89, 56.7%), PRISMA 2020 checklist was used followed by PRISMA 2009 (*n* = 26, 16.6%). Overall, 95 of the 188 included SRs either (i) cited outdated references for the reporting checklist they used (*n* = 26), (ii) used an incorrect checklist (*n* = 9), (iii) provided insufficient documentation about the reporting checklist used (*n* = 26), or (iv) did not use any reporting checklist (*n* = 34). A full list of the checklists used by the SRs is available in the supplementary material on the OSF webpage.

**Table 1:** *Descriptive statistics for the methodological, reporting, and bibliometric variables

Number of SRs included in this meta-research study	188	
Variables (continuous)	Median (IQR)	N of SRs by variable (overall)**
Journal IF (bibliometric characteristic)	3.6 (3.05)	163/188
Time from protocol registration to first submission	267 (424)	76/188
Number of individual studies included in SRs	25 (37.5)	183/188
Variables (binary)	Counts (Frequencies %)	
Funding statement (Y)	141 (75%)	188
CoI statement (Y)	181 (96.3%)	188
Data availability statement (Y)	136 (72.3%)	188
Author team > 3 (bibliometric characteristic)	144 (76.6%)	188
Non-international author team (N) (bibliometric characteristic)	135 (71.8%)	188
Registered Protocol (N)	109 (58%)	188
Reporting checklist used (Y)	154 (81.9%)	188
Reporting checklist supported (Y; N; NA)	Y: 93 (49.5%); N: 61 (32.4%); NA: 34 (18.1%)	188
RoB/methodological appraisal by two independent reviewers (Y; N; NS)	Y: 58 (31.4%); N: 7 (3.8%); NS: 120 (64.9%)	188
Title/abstract screening by two independent reviewers (Y; N; NS)	Y: 97 (51.6%); N: 12 (6.4%); NS: 79 (42%)	188
Full-text screening by two independent reviewers (Y; N; NS)	Y: 58 (30.9%); N: 15 (8%); NS: 115 (61.2%)	188
Extraction by two independent reviewers (Y; N; NS)	Y: 64 (34%); N: 24 (12.8%); NS: 100 (53.2%)	188
Meta-analysis (Y; N)	Y: 64 (34%); N: 124 (66%)	188
Certainty of evidence assessment (Y; N)	Y: 16 (8.5%); N: 172 (91.5%)	188

*CoI* Conflict of interest, *IF* Impact factor, *NA* Not applicable, *NS* Not stated, *RoB* Risk of bias, *Y * Yes

*NA in the “reported checklist supported” variable indicates that no reporting checklist was used in the SR (i.e., the authors neither report a checklist nor cite any relevant references). N indicates that the reporting checklist is not adequately supported because an incorrect checklist was used, an outdated checklist citation was provided, or documentation was insufficient (e.g., the authors state that they used a checklist but do not provide a reference). For the screening and extraction variables, N indicates that the authors explicitly state that no independent double screening/extraction was conducted, whereas NS indicates that the authors do not specify whether independent double screening/extraction was conducted

**The “N of SRs by variable (overall)” column indicates data availability for each variable (i.e., the number of included systematic reviews providing sufficient information to extract that item). Values are reported as n/188, where n is the number of SRs with extractable data for that variable. For example, journal impact factor data were available for 163 of the 188 included SRs (163/188)

Most SRs did not state whether one or more key stages of the review were conducted by two independent reviewers, specifically, the risk of bias or methodological appraisal (*n* = 120, 64.9%), full-text screening (*n* = 115, 61.2%) or data extraction (*n* = 100, 53.2%). In approximately half SRs, title/abstract screening was independently conducted by two reviewers (*n* = 97, 51.6%). Most of the included SRs did not report a meta-analysis (*n* = 124, 66%). Of 64 SRs reporting a meta-analysis, only 16 included a certainty of evidence assessment.

### Bivariate analyses of associations between key variables

Bivariate analyses showed that SRs led by authors affiliated with institutions in high-income countries were published in journals with higher IF (*W* = 1931.5, *p* < 0.0001). Similarly, SRs that included a greater number of studies were published in journals with higher IF, indicating a statistically significant, small to moderate effect size (*ρ* = 0.34, *p* < 0.0001). Additionally, SRs with a pre-registered protocol were more likely to adhere to a reporting checklist, indicating a small but statistically significant effect size (*χ*^2^ = 8.93, *p* < 0.0028; *V* = 0.22); while, SRs with more than three authors were more likely to have been pre-registered at the protocol stage, indicating a statistically significant moderate effect size (*χ*^2^ = 20.54, *p* < 0.0001; *V* = 0.34). No statistically significant associations were identified between journal IF and the existence of a pre-registered protocol, the presence of a large team, or the use of a reporting checklist. A full list of bivariate analysis results is found in Table 2.

**Table 2:** Bivariate analyses of associations between key variables

Variable 1	Variable 2	*N* of SRs included in the analysis	Test statistic*
Journal’s IF	Lead author’s affiliation with institutions in high-income countries	97	*W* = 1931.5, (*p* < 0.0002), *Sig*.
Journal’s IF	Pre-registered protocol	97	*W* = 2934.5, (*p* = 0.2326)
Journal’s IF	Author team size (> 3)	97	*W* = 2421, (*p* = 0.175)
Journal’s IF	International author team	97	*W* = 2384.5, (*p* = 0.1132)
Journal’s IF	Number of individual studies included in SRs	94	*ρ* = 0.34, (*p* < 0.0001), *Sig*.
Journal’s IF	Reporting checklist	97	*W* = 2000.5, (*p* = 0.1199)
Pre-registered protocol	Reporting checklist	188	*χ*^2^ = 8.93, *φ/V* = 0.22, (*p* < 0.0028), *Sig*.
Pre-registered protocol	Author team size (> 3)	188	*χ*^2^ = 20.54, *φ/V* = 0.34, (*p* < 0.0001), *Sig*.

*IF* Impact factor, *Sig*. Statistically significant, *V* Cramer’s V, *W* Wilcoxon rank-sum test statistic, *φ* phi coefficient, *ρ* Spearman’s rho test statistic, *χ*^*2*^ chi-squared test statistic (with Yates continuity correction)

*Estimates with *p* ≤ 0.00625 (Bonferroni-corrected) were considered statistically significant

## Discussion

To the best of our knowledge, this is the first meta-research study investigating the methodological, reporting, and bibliometric characteristics of SRs that explicitly acknowledge use of LLM-based and non-LLM automation tools in a designated statement. This study is also the first to explore associations between these SR characteristics and bibliometric characteristics, while estimating the median duration from protocol registration to first journal submission. Our findings are timely given the ongoing development of guidance on responsible and transparent AI use in evidence synthesis (including RAISE), and they provide an early empirical snapshot of how designated AI statements are being used in practice.

Across our sample, authors appeared relatively conservative in how they reported using LLM-based tools. Such as the use was predominantly described as supporting writing-related tasks, with limited reporting of tool validation, prompt sharing, and task-level descriptions. Combined with the frequent use of formulaic, near-uniform statements about human oversight and validation, these patterns raise concerns about whether current disclosures enable readers, peer reviewers, and editors to appraise the robustness, reproducibility, and potential bias introduced by AI-supported steps. This aligns with broader observations in applied natural language processing (NLP) practices in healthcare showing that reporting and validation of these practices are often inconsistent and insufficient [23]. This becomes apparent in the context of SRs, taking into account the 2025 joint position statement from Cochrane, the Campbell Collaboration, JBI and the Collaboration for Environmental Evidence that stresses the need for SR authors to transparently report all the process of any AI tool use and permeate the conduct of SR plus all the steps taken for human oversight [24].

These findings also have direct implications for academic publishers and journals. Elsevier emerged as an early adopter and dominant publisher for SRs containing explicit AI-use statements. While this may reflect proactive editorial policies and/or accelerated adoption of AI disclosure requirements, it also warrants more critical consideration: designated AI statements may function partly as publisher-level risk-management (e.g., legal/ethical safeguards) rather than purely as mechanisms for scientific transparency. The observed concentration therefore raises broader questions about cross-publisher variability in disclosure standards and peer review expectations, and whether including a designated statement is being treated as a compliance checkbox versus a meaningful contribution to reproducibility.

To note, although our results suggest relatively conservative reported use (largely writing-focussed/proof reading), this does not necessarily contradict the notion that AI will expedite the evidence synthesis process per se. A plausible interpretation is that we are observing an early adoption phase in which (i) disclosure norms are emerging faster than methodological integration, and/or (ii) AI may be used more extensively than reported but not consistently disclosed (particularly where no designated statement is required, where AI support is embedded in tools not explicitly labelled as ‘AI’, or where LLM prompts used in previous projects have been extensively modified by the researcher). In other words, conservative reported use in designated statements may coexist with increasing actual uptake over time, especially as tools mature beyond writing assistance into upstream SR tasks (search support, screening, extraction, and quality assessment).

Regarding bibliometric characteristics, SRs led by authors affiliated with high-income settings and more populated SRs in terms of included studies were more likely to appear in higher-impact journals. Additionally, larger author teams are more likely to have registered the protocols of their reviews, while reviews with pre-registered protocols are more likely to conform to a reporting checklist. In contrast, several hypothesised associations were not supported. These null findings should be interpreted cautiously because some analyses were necessarily restricted to subsets with available bibliometric data, reducing sample size and potentially limiting statistical power to detect small-to-moderate effects. Conceptually, they may also suggest that markers of transparency (e.g., protocol registration, checklist reporting) do not automatically translate into bibliometric advantage, possibly reflecting editorial and publication practices that privilege novelty over transparent reporting.

Although most SRs reported using a reporting checklist, a sizeable minority exhibited checklist-related reporting issues (e.g., outdated citations, incorrect checklist identification, or insufficient documentation). These issue types have different implications and can arise for reasons other than superficiality (e.g., journal word limits, uncertainty about checklist choice for emerging review types, or simple error). Although the occurrence of performative bias in relation to the actual use of reporting checklists cannot be precluded, we cannot support this interpretation given the limitations of our data and our screening process. Staying closer to our data and the exploratory nature of this meta-research study, these findings are better interpreted as signals of reporting limitations that may reduce the utility of checklists for readers and peer reviewers, rather than as evidence of deliberate misrepresentation.

Furthermore, several core SR processes (e.g., risk of bias assessment and data extraction) were rarely reported as being conducted independently by two reviewers. While one interpretation is that some SRs may be adopting streamlined workflows, alternative explanations are equally plausible and should be acknowledged. For instance, these patterns may reflect resource constraints alongside transparent reporting. When considered alongside our estimated median time from protocol registration to first submission, these patterns raise concerns about whether some LLM-supported SRs are associated with shorter timelines. However, this finding should be interpreted with caution given missing data for some impact factors, as well as the way we approximated the time lag from protocol registration to submission when submission dates were unavailable (i.e., using the mean time lag reported by the journal from first submission to publication). Reported LLM use was primarily writing-focussed, and the degree to which AI contributes to speed (versus other factors) remains uncertain and is a priority for future comparative meta-research.

### Strengths and limitations

This meta-research study exhibits some key strengths. First, it was based on a pre-registered protocol registered with OSF. Second, it is the first meta-research study that focusses on the methodological, reporting and bibliometric characteristics of those SRs that have explicitly acknowledged the use of LLM-based and non-LLM automation tools in a designated publication statement.

However, this study inevitably includes some limitations. First, the SRs were identified using targeted Google Scholar searches, with the possibility that some SRs containing designated AI statements were missed because such statements are inconsistently indexed and retrievability depends on Google Scholar’s coverage and ranking algorithms. Second, although the final sample was randomly selected within a purposively defined stratum (i.e., records retrieved via these targeted queries), the purposive definition of that stratum may itself introduce selection bias, taking also into account that a number of potentially eligible SRs were not able to be retrieved; therefore, our findings should be interpreted as characterising SRs captured by this targeted identification approach rather than being fully generalisable to all SRs using LLMs and/or non-LLM automation tools. Third, we only included SRs published in English, potentially excluding relevant reviews published in other languages. Fourth, in certain instances, journal impact factors were unavailable due to journals not being indexed, and submission dates were occasionally missing. In cases of missing submission dates, we used approximations by subtracting the mean number of days from the first submission to publication, based on information provided by the respective journal’s website; these analyses should therefore be interpreted cautiously, as the approximation assumes relative stability in journal processing times and may not hold for all manuscripts.

### Implications for practice and research

Our findings underscore the urgency of developing reporting standards and guidelines specifically tailored to LLMs’ use in SRs. These results have implications for academic journals and publishers, suggesting the need to mandate clearer AI disclosures, including prompt sharing, detailed task-level descriptions, and transparent reporting of human oversight (including concrete verification procedures). Publishers and journals should establish consistent policies regarding how AI is integrated and reported in systematic reviews and should consider whether designated AI statements are being implemented as meaningful transparency mechanisms versus minimal compliance artefacts. Future meta-research should compare LLM-supported SRs with SRs not disclosing AI use (or disclosing it differently), including whether timelines differ, and which SR tasks are most affected, while explicitly accounting for potential identification (e.g., sampling bias) introduced by disclosure-dependent retrieval.

## Supplementary Information

Supplementary Material 1.

## Data Availability

Supplementary data and materials are available on the OSF webpage for this study (https://osf.io/uy2pc).

## Footnotes

1
https://harzing.com/resources/publish-or-perish.
