# Supplementary material for: Bibliometric, methodological and reporting characteristics of systematic reviews with explicit AI disclosure statements: an exploratory meta-research study
Source: BMC Med Res Methodol. 2026 Feb 11;26:59. doi: 10.1186/s12874-026-02796-2 (PMC12997672; doi:10.1186/s12874-026-02796-2)
Supplement: Supplementary file 1 — Supplementary Material 1. [file 12874_2026_2796_MOESM1_ESM.docx]

**Supplementary material**

**Title:** Bibliometric, methodological and reporting characteristics of systematic reviews with explicit AI disclosure statements: an exploratory meta-research study

**Authors:** Bastounis, A., Lagojda, L., Sheppard, W., Daly, G., Poku, E. & Booth, A.

**Registration:** <https://osf.io/uy2pc>

**Appendix I:** List of eligibility criteria

**Appendix II:** Combinations of search terms used and adapted PRISMA flow diagram

**Appendix III:** Sunburst chart of LLM-based and non-LLM automation tools included in this meta-research study

**Appendix IV:** PRISMA 2020 reporting checklist (completed)

**Appendix V:** List of systematic reviews included in the meta-research study

**Appendix I**

**Table 1.** List of eligibility criteria

| **Categories** | **Inclusion criteria** | **Exclusion criteria** |
| --- | --- | --- |
| Population | All populations | None |
| Intervention/phaenomenon of interest | Eligible SRs were required to include an explicit mention of the use of assisted-AI tools and/or LLMs (e.g., ChatGPT, Claude, Gemini, or other large language models) in at least one of the title, abstract, author list, or AI-use specific supporting statement. | Systematic reviews where AI was only used in included studies, not in the conduct of the SR itself.  Systematic reviews that omitted a designated statement regarding the use of AI for SRs and those that have used mainstream review management software that incorporates AI tools (i.e., Rayyan, Covidence). |
| Control conditions | Not applicable | Not applicable |
| Outcomes | Meta-data (i.e., bibliometric, reporting and methodological characteristics, meta-data related to AI-use and human validation) | Clinical or any data not related to meta-research |
| Study design | Systematic reviews, including methodological reviews, diagnostic test accuracy reviews, and network meta-analyses.  Systematic reviews labelled as “scoping reviews” but their authors had extended a systematic approach to multiple stages of the review (i.e., searching, screening, extraction, risk-of-bias, methodological appraisal) in addressing a clearly-demarcated research question. | Narrative, scoping, and rapid reviews. Overviews of reviews (i.e., umbrella reviews). |
| Language | Only English | Non-English |
| Setting | All scientific disciplines, topics, and geographical contexts | None |
| Publication type | Manuscripts published in peer-reviewed journals | Editorials, commentaries, letters, conference abstracts, protocols, theses/dissertations, preprints |

**Appendix II**

**Table 2.** Combinations of search terms used, and number of records retrieved for each combination.

| **Search combinations** | **Number of records** |
| --- | --- |
| systematic review [title] AND "AI Statement:" | 568 |
| systematic review AND "Use of Generative AI" | 172 |
| "systematic review" AND "Declaration of Generative AI" | 560 |
| "systematic review" AND "Declaration of Generative Artificial Intelligence" | 3 |
| "systematic review" AND "Use of Generative Artificial Intelligence" | 58 |

**Figure 1.** Adapted version of the PRISMA flow diagram


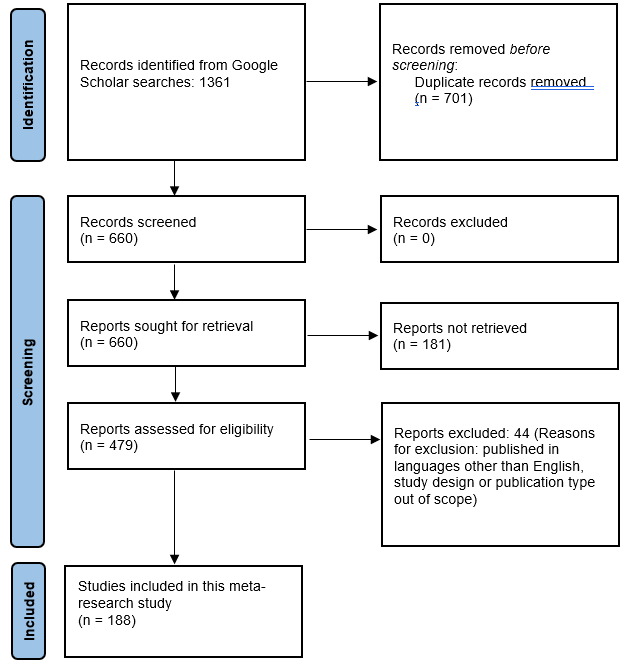


*Study selection process*

Overall, 1,361 records were identified through searches in Google Scholar using highly precise search combination terms. Of these, 701 duplicates were identified in Excel and removed. From the remaining 660 records, full texts for 181 could not be retrieved. In total, 479 records were assessed at the full-text stage, with 44 excluded predominantly because the study design and publication type were out of scope. From the 435 potentially eligible records, a random sample of 188 studies was selected and included in this meta-research study.

**Appendix III**

| **Figure 1**. Sunburst chart displaying the proportion of LLM-based and non-LLM automation tools used in the included SRs. The inner ring represents the AI tool, and the outer ring shows the corresponding review stage wherein the tools was used. |
| --- |
| 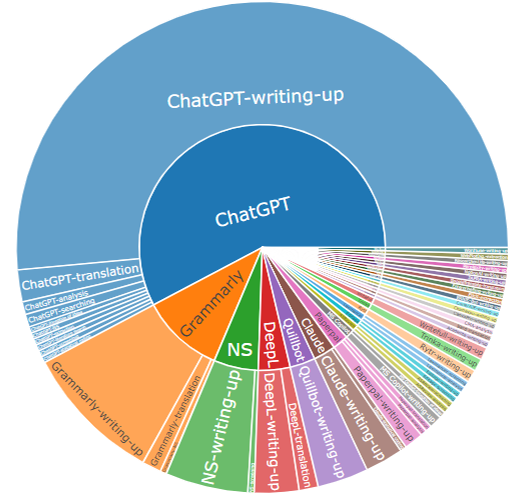 |

**Appendix IV**

**Table 3.** PRISMA 2020 reporting checklist (Page et al., 2021)

| **Section and Topic** | **Item #** | **Checklist item** | **Location where item is reported** |
| --- | --- | --- | --- |
| **TITLE** | | |  |
| Title | 1 | Identify the report as a systematic review. | 1 |
| **ABSTRACT** | | |  |
| Abstract | 2 | See the PRISMA 2020 for Abstracts checklist. | 1 |
| **INTRODUCTION** | | |  |
| Rationale | 3 | Describe the rationale for the review in the context of existing knowledge. | 2,3 |
| Objectives | 4 | Provide an explicit statement of the objective(s) or question(s) the review addresses. | 3 |
| **METHODS** | | |  |
| Eligibility criteria | 5 | Specify the inclusion and exclusion criteria for the review and how studies were grouped for the syntheses. | 3,4 (plus supplementary material) |
| Information sources | 6 | Specify all databases, registers, websites, organisations, reference lists and other sources searched or consulted to identify studies. Specify the date when each source was last searched or consulted. | 4 |
| Search strategy | 7 | Present the full search strategies for all databases, registers and websites, including any filters and limits used. | 4 (plus supplementary material) |
| Selection process | 8 | Specify the methods used to decide whether a study met the inclusion criteria of the review, including how many reviewers screened each record and each report retrieved, whether they worked independently, and if applicable, details of automation tools used in the process. | 4 (plus supplementary material) |
| Data collection process | 9 | Specify the methods used to collect data from reports, including how many reviewers collected data from each report, whether they worked independently, any processes for obtaining or confirming data from study investigators, and if applicable, details of automation tools used in the process. | 4 |
| Data items | 10a | List and define all outcomes for which data were sought. Specify whether all results that were compatible with each outcome domain in each study were sought (e.g. for all measures, time points, analyses), and if not, the methods used to decide which results to collect. | 4,5 |
|  | 10b | List and define all other variables for which data were sought (e.g. participant and intervention characteristics, funding sources). Describe any assumptions made about any missing or unclear information. | 4,5 |
| Study risk of bias assessment | 11 | Specify the methods used to assess risk of bias in the included studies, including details of the tool(s) used, how many reviewers assessed each study and whether they worked independently, and if applicable, details of automation tools used in the process. | N/A (this is a meta-research study) |
| Effect measures | 12 | Specify for each outcome the effect measure(s) (e.g. risk ratio, mean difference) used in the synthesis or presentation of results. | 5 (statistical tests and types of data analysed – meta-research study) |
| Synthesis methods | 13a | Describe the processes used to decide which studies were eligible for each synthesis (e.g. tabulating the study intervention characteristics and comparing against the planned groups for each synthesis (item #5)). | 5 (statistical analysis) |
|  | 13b | Describe any methods required to prepare the data for presentation or synthesis, such as handling of missing summary statistics, or data conversions. | N/A (this is a meta-research study) |
|  | 13c | Describe any methods used to tabulate or visually display results of individual studies and syntheses. | 5 |
|  | 13d | Describe any methods used to synthesize results and provide a rationale for the choice(s). If meta-analysis was performed, describe the model(s), method(s) to identify the presence and extent of statistical heterogeneity, and software package(s) used. | N/A (this is a meta-research study) |
|  | 13e | Describe any methods used to explore possible causes of heterogeneity among study results (e.g. subgroup analysis, meta-regression). | N/A (this is a meta-research study) |
|  | 13f | Describe any sensitivity analyses conducted to assess robustness of the synthesized results. | N/A (this is a meta-research study) |
| Reporting bias assessment | 14 | Describe any methods used to assess risk of bias due to missing results in a synthesis (arising from reporting biases). | N/A (this is a meta-research study) |
| Certainty assessment | 15 | Describe any methods used to assess certainty (or confidence) in the body of evidence for an outcome. | N/A (this is a meta-research study) |
| **RESULTS** | | |  |
| Study selection | 16a | Describe the results of the search and selection process, from the number of records identified in the search to the number of studies included in the review, ideally using a flow diagram. | 6 (plus supplementary material) |
|  | 16b | Cite studies that might appear to meet the inclusion criteria, but which were excluded, and explain why they were excluded. | N/A (a random sample was drawn from a purposively defined stratum of eligible studies) |
| Study characteristics | 17 | Cite each included study and present its characteristics. | Supplementary material |
| Risk of bias in studies | 18 | Present assessments of risk of bias for each included study. | N/A (this is a meta-research study) |
| Results of individual studies | 19 | For all outcomes, present, for each study: (a) summary statistics for each group (where appropriate) and (b) an effect estimate and its precision (e.g. confidence/credible interval), ideally using structured tables or plots. | Supplementary material |
| Results of syntheses | 20a | For each synthesis, briefly summarise the characteristics and risk of bias among contributing studies. | 6,7 |
|  | 20b | Present results of all statistical syntheses conducted. If meta-analysis was done, present for each the summary estimate and its precision (e.g. confidence/credible interval) and measures of statistical heterogeneity. If comparing groups, describe the direction of the effect. | N/A (this is a meta-research study) |
|  | 20c | Present results of all investigations of possible causes of heterogeneity among study results. | N/A (this is a meta-research study) |
|  | 20d | Present results of all sensitivity analyses conducted to assess the robustness of the synthesized results. | N/A (this is a meta-research study) |
| Reporting biases | 21 | Present assessments of risk of bias due to missing results (arising from reporting biases) for each synthesis assessed. | N/A (this is a meta-research study) |
| Certainty of evidence | 22 | Present assessments of certainty (or confidence) in the body of evidence for each outcome assessed. | N/A (this is a meta-research study) |
| **DISCUSSION** | | |  |
| Discussion | 23a | Provide a general interpretation of the results in the context of other evidence. | 10 - 12 |
|  | 23b | Discuss any limitations of the evidence included in the review. | 12 |
|  | 23c | Discuss any limitations of the review processes used. | 12 |
|  | 23d | Discuss implications of the results for practice, policy, and future research. | 13 |
| **OTHER INFORMATION** | | |  |
| Registration and protocol | 24a | Provide registration information for the review, including register name and registration number, or state that the review was not registered. | 3, 13 |
|  | 24b | Indicate where the review protocol can be accessed, or state that a protocol was not prepared. | 3, 13 |
|  | 24c | Describe and explain any amendments to information provided at registration or in the protocol. | N/A |
| Support | 25 | Describe sources of financial or non-financial support for the review, and the role of the funders or sponsors in the review. | 13 |
| Competing interests | 26 | Declare any competing interests of review authors. | 13 |
| Availability of data, code and other materials | 27 | Report which of the following are publicly available and where they can be found: template data collection forms; data extracted from included studies; data used for all analyses; analytic code; any other materials used in the review. | 13 |

**Appendix V**

List of included systematic reviews included in the meta-research study

1. Banerjee S, Das T, Chaubey G, Srivastava, A. . Unveiling the diversity and potential of STR markers across India - a systematic review. Human Gene 2025.

2. Labrague LJ, Al Harrasi M. Nursing students' perceptions of artificial intelligence (AI) using the technology acceptance model: A systematic review. Teaching and Learning in Nursing. 2025;20:274-82.

3. Dashti M, Khosraviani F, Azimi T, Sehat MS, Alekajbaf E, Fahimipour A, et al. Predicting Mandibular Bone Growth Using Artificial Intelligence and Machine Learning: A Systematic Review. Advances in Artificial Intelligence and Machine Learning. 2024;4(3).

4. Nikolic S, Wentworth I, Sheridan L, Moss S, Duursma E, Jones RA, et al. A systematic literature review of attitudes, intentions and behaviours of teaching academics pertaining to AI and generative AI (GenAI) in higher education: An analysis of GenAI adoption using the UTAUT framework. Australasian Journal of Educational Technology. 2024;40(6).

5. Zhang Υ, Li Η, Numtong ΚT, Ν., Ran Μ. Systematic literature review on the heritage language teachers agency from micro Perspective. Journal ofThai-Chinese Social Science. 2024;1(1).

6. Abdullahi S, Yang Z, Hassan Gama MI, Mohammed Omer MO, Wang Q, Yakubu A, et al. Enhancing the sensitivity and accuracy of wearable glucose biosensors: A systematic review on the prospects of mutarotase. Sensors and Actuators Reports. 2024;8.

7. Ackah M, Barakou I, Abonie US, Hettinga FJ. Adherence to exercise in breast cancer survivors during and after active treatment: A systematic review and meta-analysis. JSAMS Plus. 2024;4.

8. Aghaloo K, Sharifi A, Habibzadeh N, Ali T, Chiu Y-R. How nature-based solutions can enhance urban resilience to flooding and climate change and provide other co-benefits: A systematic review and taxonomy. Urban Forestry & Urban Greening. 2024;95.

9. Ahmadi N, Alirezaei F, Mohaghegh S, Kouhestani F, Motamedian SR. Application of Stem Cells in Consolidation Phase of Distraction Osteogenesis: A Systematic Review and Meta-Analysis. Journal of Maxillofacial and Oral Surgery. 2025.

10. Ahmadi-Hadad A, de Queiroz PCC, Schettini F, Giuliano M. Reawakening the master switches in triple-negative breast cancer: A strategic blueprint for confronting metastasis and chemoresistance via microRNA-200/205: A systematic review. Crit Rev Oncol Hematol. 2024;204:104516.

11. Ahmed A, Watterson C, Alhashmi S, Gaber T. How universities teach cybersecurity courses online: a systematic literature review. Frontiers in Computer Science. 2024;6.

12. Albuquerque DAN, Vianna MT, Sampaio LAF, Vasiliu A, Neves Filho EHC. Systematic review and meta-analysis of artificial intelligence in classifying HER2 status in breast cancer immunohistochemistry. NPJ Digit Med. 2025;8(1):144.

13. Alcorn M, Koo B. Wellness dimensions of hospitality employees: A systematic review. International Journal of Hospitality Management. 2025;124.

14. Alemu BK, Wang CC, Li L, Zhu Z, Li Q, Wang Y. Effect of preconception antibiotics exposure on female reproductive health and pregnancy outcomes: a systematic review and meta-analysis. EClinicalMedicine. 2024;78:102935.

15. Alimam W, Auvinen A. Cancer risk due to ingestion of naturally occurring radionuclides through drinking water: A systematic review. Sci Total Environ. 2025;968:178849.

16. Alnasyan B, Basheri M, Alassafi M. The power of Deep Learning techniques for predicting student performance in Virtual Learning Environments: A systematic literature review. Computers and Education: Artificial Intelligence. 2024;6.

17. Alshamrani A, Aznar M, Hoskin P, Chuter R, Eccles CL. The Current use of Adaptive Strategies for External Beam Radiotherapy in Cervical Cancer: A Systematic Review. Clin Oncol (R Coll Radiol). 2024;36(12):e483-e93.

18. Alvarez-Lozada LA, Fernandez-Reyes BA, Arrambide-Garza FJ, Garcia-Leal M, Alvarez-Villalobos NA, Martinez-Garza JH, et al. Clinical scores for acute appendicitis in adults: A systematic review and meta-analysis of diagnostic accuracy studies. Am J Surg. 2025;240:116123.

19. Amirsardari Z, Amirsardari F, Kohansal E, Jolfay AG, Dehaki MG, Ziaee V. Exploring the association between serum Vitamin D levels and the development of coronary artery lesions in Kawasaki disease - a systematic review. Pediatr Rheumatol Online J. 2024;22(1):71.

20. Amo-Filva D, Fonseca D, García-Peñalvo FJ, Forment MA, Casany Guerrero MJ, Godoy G. Exploring the landscape of learning analytics privacy in fog and edge computing: A systematic literature review. Computers in Human Behavior. 2024;158.

21. Anton E, Zurera-Egea C, Farriol R, Sarrate Z, Blanco J. Systematic review and evidence-based classification of differentially expressed mRNAs in human sperm: insights to improve male infertility diagnosis and prognosis. Reproductive BioMedicine Online. 2025.

22. Askarinejad A, Arya A, Zangiabadian M, Ghahramanipour Z, Hesami H, Farmani D, et al. Catheter ablation as first-line treatment for ventricular tachycardia in patients with structural heart disease and preserved left ventricular ejection fraction: a systematic review and meta-analysis. Sci Rep. 2024;14(1):18536.

23. Askarinejad A, Kohansal E, Sabahizadeh A, Hesami H, Adimi S, Haghjoo M. Pulsed-Field Ablation in Management of Ventricular Tachycardia: A Systematic Review of Case Reports and Clinical Outcomes. Clin Cardiol. 2024;47(10):e70018.

24. Bangash AH, Ryvlin J, Fourman MS, Gelfand Y, Murthy SG, Yassari R, et al. Safety and efficacy of tranexamic acid in oncologic surgery for spinal metastases: A systematic review. North American Spine Society Journal (NASSJ). 2025;22.

25. Bernasconi A, Landi M, Yah CS, van der Sande MAB. Information and Communication Technology to Enhance the Implementation of the Integrated Management of Childhood Illness: A Systematic Review and Meta-Analysis. Mayo Clin Proc Digit Health. 2024;2(3):438-52.

26. Bérubé C, Nißen M, Vinay R, Geiger A, Budig T, Bhandari A, et al. Proactive behavior in voice assistants: A systematic review and conceptual model. Computers in Human Behavior Reports. 2024;14.

27. Beste ME, Kaunitz AM, McKinney JA, Sanchez-Ramos L. Vaginal estrogen use in breast cancer survivors: a systematic review and meta-analysis of recurrence and mortality risks. Am J Obstet Gynecol. 2025;232(3):262-70 e1.

28. Bhatnagar A, Härri A, Levänen J, Niinimäki K. Exploring the role of social life cycle assessment in transition to circular economy: A systematic review. Resources, Conservation and Recycling. 2024;207.

29. Bozkurt SA, Aydogan S, Dursun Ergezen F, Turkoglu A. A systematic review and sequential explanatory synthesis: Artificial intelligence in healthcare education, a case of nursing. Int Nurs Rev. 2025;72(2):e70018.

30. Burström T, Lahti T, Parida V, Wincent J. Industrial ecosystems: A systematic review, framework and research agenda. Technological Forecasting and Social Change. 2024;208.

31. Candreva A, Buongiorno AL, Matter MA, Rizzini ML, Giacobbe F, Ravetti E, et al. Impact of endothelial shear stress on coronary atherosclerotic plaque progression and composition: A meta-analysis and systematic review. Int J Cardiol. 2024;407:132061.

32. Cavero B, Martinez-Castilla P, Campos R. Let's make music as we normally do: A systematic review of how early natural musical interactions between infant and caregiver have been studied in research. Infant Behav Dev. 2024;75:101928.

33. Chiaranai C, Chularee S, Doommai N, Liangchawengwong S. Understanding the Lived Experience of Patients With Heart Failure During the COVID-19 Pandemic: A Systematic Review and Meta-synthesis of Qualitative Studies. J Cardiovasc Nurs. 2025.

34. Chiaranai C, Chularee S, Saokaew S, Bhatarasakoon P, Umnuaypornlert A, Chaomuang N, et al. Effectiveness of telehealth on the glycemic control of patients with type 2 diabetes mellitus during the COVID-19 pandemic: A systematic review and meta-analysis of randomised controlled trials. Int J Nurs Stud Adv. 2024;6:100169.

35. Chiu YT, Chen YT, Lee FJ, Chang CY. The impact of glucagon-like peptide-1 receptor agonists on the quality indicators of colonoscopy - a systematic review and meta-analysis. Dig Liver Dis. 2025;57(7):1386-92.

36. Conde MÁ, Rodríguez-Sedano FJ. Is learning analytics applicable and applied to education of students with intellectual/developmental disabilities? A systematic literature review. Computers in Human Behavior. 2024;155.

37. Cortese N, Procopio A, Merola A, Zaffino P, Cosentino C. Applications of genome-scale metabolic models to the study of human diseases: A systematic review. Comput Methods Programs Biomed. 2024;256:108397.

38. Crestois N, Kempton MJ, Twumasi R. A systematic review and meta-analysis of employer discrimination towards people living with psychosis. Schizophr Res. 2025;278:35-46.

39. Dashti M, Azimi T, Khosraviani F, Azimian S, Bahanan L, Zahmatkesh H, et al. Systematic Review and Meta-Analysis on the Accuracy of Artificial Intelligence Algorithms in Individuals Gender Detection Using Orthopantomograms. Int Dent J. 2025;75(3):2157-68.

40. Dashtkoohi M, Ghadimi DJ, Moodi F, Behrang N, Khormali E, Salari HM, et al. Focal cortical dysplasia detection by artificial intelligence using MRI: A systematic review and meta-analysis. Epilepsy Behav. 2025;167:110403.

41. Decabooter I, Warmoes A, Consuegra E, Van Gasse R, Struyven K. The data coach chronicles: A systematic review of human support for making data-based decision-making a success. Teaching and Teacher Education. 2024;146.

42. Deepika J, Vijayalakshmi D, Aravind Kumar S, Vinitha A, Ravichandran V, Vellaikumar S, et al. A systematic literature review on current status and future prospects of pre-harvest sprouting management in rice. Plant Science Today. 2024.

43. Denis C, Boucaud-Maitre D, Brunelin J, Jurek L, Vallet W, Demily C. Prevalence of attention deficit hyperactivity disorder in homeless children and adolescents: A systematic review and meta-analysis. Dialogues Clin Neurosci. 2025;27(1):86-97.

44. Dilokthornsakul P, Rattanachaisit N, Thimkorn P, Pongpattanawut S, Dilokthornsakul W, Dhippayom T. Clinical effects of Hibiscus sabdariffa Linn. on obesity treatment: A systematic review and meta-analysis of randomized controlled trials. Complement Ther Med. 2024;84:103063.

45. Ebrahimi F, Masoudian T, Khiabani MM. Integrating ADDIE Needs Assessment with Kirkpatrick Evaluation: A Systematic Review. Asian Journal of Education and Social Studies. 2025;51(3):350-76.

46. Ellwood J, Kovanur Sampath K, Rund I, Treffel L, Draper-Rodi J. Guidance strategies for infantile asymmetry prevention: a systematic review. BMC Pediatr. 2025;25(1):328.

47. Enchikova E, Neves T, Toledo C, Nata G. Change in socioeconomic educational equity after 20 years of PISA: A systematic literature review. International Journal of Educational Research Open. 2024;7.

48. Erakca M, Baumann M, Helbig C, Weil M. Systematic review of scale-up methods for prospective life cycle assessment of emerging technologies. Journal of Cleaner Production. 2024;451.

49. Erbicer ES, Boranli EN, Metin A, Erbicer S, Sen S, Demirtas ET, et al. Cyber Dating Violence Among Youth and Adolescents: A Systematic Review and Meta-Analysis. J Youth Adolesc. 2025;54(3):625-48.

50. Errazuriz A, Avello-Vega D, Passi-Solar A, Torres R, Bacigalupo F, Crossley NA, et al. Prevalence of anxiety disorders in Latin America: a systematic review and meta-analysis. Lancet Reg Health Am. 2025;45:101057.

51. Eumi EM. A systematic review of Digital Twins in efficient pandemic management with challenges and emerging trends. Decision Analytics Journal. 2024;12.

52. Exner B, Frielitz-Wagner IV, Frielitz FS. Telemedicine and digital health for chronic conditions in pediatrics: A systematic review. J Telemed Telecare. 2025:1357633X251334423.

53. Francis NS, Lim YM, Mat S, Loganathan A. Effectiveness of herbs taken concurrently with antihypertensive drugs in managing hypertension and lipid outcomes. A systematic review and meta-analysis. Complement Ther Med. 2024;83:103058.

54. Frivaldszky L, Lorincz K, Hoferica J, Hegyi P, Acs N, Melczer Z, et al. Esketamine reduces the risk of postpartum depression in women undergoing cesarean section: A systematic review and meta-analysis. J Psychiatr Res. 2025;183:164-73.

55. Gao W, Larjavaara M. Wind disturbance in forests: A bibliometric analysis and systematic review. Forest Ecology and Management. 2024;564.

56. Garroussi Z, Legrain A, Gambs S, Gautrais V, Sansò B. A systematic review of data privacy in Mobility as a Service (MaaS). Transportation Research Interdisciplinary Perspectives. 2025;31.

57. Geck S, Roithmeier M, Buhner M, Wehr S, Weigel L, Priller J, et al. COSMIN systematic review and meta-analysis of the measurement properties of the Positive and Negative Syndrome Scale (PANSS). EClinicalMedicine. 2025;82:103155.

58. Gellisch M, Bablok M, Brand-Saberi B, Schafer T. Neurobiological stress markers in educational research: A systematic review of physiological insights in health science education. Trends Neurosci Educ. 2024;37:100242.

59. Ghadimi N, Daroudi R, Shabaninejad H, Goharimehr M, Khodamorzideh D, Kaveh S. Cost-effectiveness of Evolocumab in Cardiovascular Disease: A Systematic Review. Curr Ther Res Clin Exp. 2024;101:100758.

60. Ghodeswar A, Bhandari M, Hedman B. Quantifying the economic costs of power outages owing to extreme events: A systematic review. Renewable and Sustainable Energy Reviews. 2025;207.

61. Ghorbani M, Abouei Mehrizi M, Tajvidi M, Amin Habibi M, Mohammadi M, Esmaeilian S, et al. Trehalose: A promising new treatment for traumatic brain injury? A systematic review of animal evidence. Interdisciplinary Neurosurgery. 2024;36.

62. Goddard RJ, Krijnen WP, Roelfsema V, Waninge A, Driessen JMM. The prevalence of bruxism in children with profound intellectual and multiple disabilities; a systematic review and meta-analysis. Brain Disorders. 2024;15.

63. Golmohammadi M, Samadi M, Salimi Y, Nachvak SM, Ebrahimzadeh Attari V. The association of dietary inflammatory index with sleep outcomes: A systematic review. Health Promot Perspect. 2024;14(2):136-47.

64. Gómez JM, Fares OH, Mohan M, Lee SH. Blockchain in the Food Industry: Integrating Machine Learning in a Systematic Literature Review. Journal of International Technology and Information Management. 2023;32(1):32-58.

65. González A, Quibano-Ordoñez D, Ortega-Muñoz L, Moreno PA, Vélez-Varela PE. Genetic variants in the development of autoimmune complaints and capsular contracture in women with breast implants: A systematic review. JPRAS Open. 2025;44:529-41.

66. Gottardo A, Gristina V, Perez A, Di Giovanni E, Contino S, Barraco N, et al. Roles of Tumor-Educated Platelets (TEPs) in the biology of Non-Small Cell Lung Cancer (NSCLC): A systematic review. "Re-discovering the neglected biosources of the liquid biopsy family". J Liq Biopsy. 2024;3:100136.

67. Gou J, Li J, Li Y, Lu M, Wang C, Zhuo Y, et al. The Diagnostic Accuracy Between Radiomics Model and Non-radiomics Model for Preoperative of Microvascular Invasion of Solitary Hepatocellular Carcinoma: A Systematic Review and Meta-analysis. Acad Radiol. 2024;31(11):4419-33.

68. Guerrero-Ocampo M, Tagle-Vega U, Flecha-Salgueiro I, Riquelme A, Diaz D, Frutos F. Feasibility of simultaneous meningioma resection and cesarean section: A systematic review and a technical case presentation. World Neurosurg X. 2025;25:100417.

69. Gunawan J, Aungsuroch Y, Montayre J. ChatGPT integration within nursing education and its implications for nursing students: A systematic review and text network analysis. Nurse Educ Today. 2024;141:106323.

70. HajiEsmailPoor Z, Kargar Z, Tabnak P. Radiomics diagnostic performance in predicting lymph node metastasis of papillary thyroid carcinoma: A systematic review and meta-analysis. Eur J Radiol. 2023;168:111129.

71. Haque MN, Sharifi A. Who are marginalized in accessing urban ecosystem services? A systematic literature review. Land Use Policy. 2024;144.

72. Hardi H, Estuworo GK, Louisa M. Effectivity of oral ginger supplementation for chemotherapy induced nausea and vomiting (CINV) in children: A systematic review of clinical trials. J Ayurveda Integr Med. 2024;15(4):100957.

73. Horstman A, Smith JAS, Bassed RB, Bugeja L. The impacts on paediatricians testifying in cases of child maltreatment: A systematic scoping review. Child Abuse Negl. 2025;163:107357.

74. Huerta MA, Cisneros E, Alique M, Roza C. Strategies for measuring non-evoked pain in preclinical models of neuropathic pain: Systematic review. Neurosci Biobehav Rev. 2024;163:105761.

75. Huerta MA, Marcos-Frutos D, Nava J, Garcia-Ramos A, Tejada MA, Roza C. P2X3 and P2X2/3 receptors inhibition produces a consistent analgesic efficacy: A systematic review and meta-analysis of preclinical studies. Eur J Pharmacol. 2024;984:177052.

76. Ikrama M, Usama M, Israr S, Humayon M, Umair Javaid M, Abbas S, et al. Future of fibrodysplasia ossificans progressiva management: A systematic review of investigational therapies. Rheumatology & Autoimmunity. 2025;5(1):28-36.

77. Isaji Y, Kurasawa Y, Sasaki D, Hayashi M, Kitagawa T. Psychological intervention for knee osteoarthritis: a systematic review and meta-analysis. Psychol Health Med. 2025;30(3):636-62.

78. Ismail UN, Yahya N, Manan HA. Investigating functional connectivity related to stroke recovery: A systematic review. Brain Res. 2024;1840:149023.

79. Jachmann A, Loser A, Mettler A, Exadaktylos A, Muller M, Klingberg K. Burnout, Depression, and Stress in Emergency Department Nurses and Physicians and the Impact on Private and Work Life: A Systematic Review. J Am Coll Emerg Physicians Open. 2025;6(2):100046.

80. Jager LAd, Claassen L, Scholz G, Chappin EJL, van Bruggen A. Household heat pump adoption and user behaviours: a systematic review of drivers and barriers. International Journal of Sustainable Energy. 2025;44(1).

81. Jang S, Park J, Yu G, Hwang J. Tools for estimating traffic emissions considering spatial scales and mitigation strategies: A systematic review. International Journal of Sustainable Transportation. 2025;19(5):431-45.

82. Jerez Yañez O, Kim J, Bonilla Mejia J, Montalvo F, Veas Romero M. Perspectivas Estudiantiles y Docentes sobre la IA en la Educación Sanitaria: Revisión Sistemática Exploratoria. Revista Española de Educación Médica. 2025;6(2).

83. Kahl F, Kapsecker M, Nissen L, Bresser L, Heinemann M, Reimer LM, et al. Digital Technologies in Hereditary Coagulation Disorders: A Systematic Review. Hamostaseologie. 2024;44(6):446-58.

84. Karaman Madan Ö, Chamilothori K, van Duijnhoven J, Aarts MPJ, de Kort YAW. Restorative effects of daylight in indoor environments – A systematic literature review. Journal of Environmental Psychology. 2024;97.

85. Keles S, Munthe E. A systematic review of research on how initial teacher education prepares teachers for work on discrimination, racism and prejudices in schools. Review of Education. 2025;13(1).

86. Kelly D, Ostovar-Kermani TG. Systematic Literature Review of Disseminating Health Information to BIPOC Communities. Health Open Research. 2024;6.

87. Kernif T, Medrouh B, Eddaikra N, Oury B, Holzmuller P, Sereno D. Ticks as vectors of Trypanosomatidae with medical or veterinary interest: Insights and implications from a comprehensive systematic review and meta-analysis. Heliyon. 2024;10(24):e40895.

88. Khaqan S, Redondo-Sama G. A systematic review of the role of dialogic leadership: Characterization and impacts. Educational Research Review. 2024;44.

89. Kohansal E, Jamalkhani S, Hosseinpour A, Yousefimoghaddam F, Askarinejad A, Hekmat E, et al. Invasive versus conservative strategies for non-ST-elevation acute coronary syndrome in the elderly: an updated systematic review and meta-analysis of randomized controlled trials. BMC Cardiovasc Disord. 2025;25(1):96.

90. Kronlid C, Brantnell A, Elf M, Borg J, Palm K. Sociotechnical analysis of factors influencing IoT adoption in healthcare: A systematic review. Technology in Society. 2024;78.

91. Kuhn C, Hayibor KM, Acheampong AT, Pires LSA, Costa-Ribeiro MCV, Burrone MS, et al. How studies on zoonotic risks in wildlife implement the one health approach - A systematic review. One Health. 2024;19:100929.

92. Kurvinen M, Lamminpaa R, Vehvilainen-Julkunen K. Women's experiences of waterbirth: A systematic review with narrative synthesis. Midwifery. 2025;147:104434.

93. Lamzouri O, Taheri H, Saadi H, Mimouni A. Prophylactic Bilateral Salpingectomy in Preventing Ovarian Cancer in High Risk Women: A Systematic Review. Indian Journal of Gynecologic Oncology. 2025;23(2).

94. Landau M, Comeaux M, Mortell T, Boyle R, Imbrescia K, Chaffin AE. Characterizing the untapped potential of virtual reality in plastic and reconstructive surgical training: A systematic review on skill transferability. JPRAS Open. 2024;41:295-310.

95. Lausten PLM, Christensen VB, Kissow H. Matrix metalloproteinase 7 as a diagnostic biomarker of biliary atresia: A systematic review. Advances in Biomarker Sciences and Technology. 2024;6:72-82.

96. Li B, Lowell VL, Wang C, Li X. A systematic review of the first year of publications on ChatGPT and language education: Examining research on ChatGPT’s use in language learning and teaching. Computers and Education: Artificial Intelligence. 2024;7.

97. Li J, Yazid Z. Systematic Literature Review of Exploring the Role of Leader during Conflict: A Case of Virtual Teams. Wseas Transactions on Business and Economics. 2025;22:333-48.

98. Lieberum JL, Tows M, Metzendorf MI, Heilmeyer F, Siemens W, Haverkamp C, et al. Large language models for conducting systematic reviews: on the rise, but not yet ready for use-a scoping review. J Clin Epidemiol. 2025;181:111746.

99. Liu PP, Chang JC, Hsu JY, Huang HK, Loh CH, Yeh JI. Off-hours Surgery and Mortality in Patients With Type A Aortic Dissection Repair: A Systematic Review and Meta-Analysis. Korean Circ J. 2024;54(3):126-37.

100. Liu Y, Xu T, Tan Q, Xiong L. Effects of Candida colonization on patients with ventilator-associated pneumonia and pathogenic microorganisms: Systematic review and meta-analysis. Diagn Microbiol Infect Dis. 2025;111(1):116580.

101. Luo J, Liu X. What do we mean by digital equality in education? Toward five conceptual lenses based on a systematic review. Journal of Research on Technology in Education. 2025:1-21.

102. Mao Y, Liu F. Neural Machine Translation in Second Language Writing: a Systematic Review. International Journal of Education and Information Technologies. 2024;18:143-53.

103. Martins MV, Jolic Marjanovic Z, Ferreira N, Hancheva C, Motrico E, Mestre JM, et al. Evaluation of assessment instruments for working alliance in psychological interventions with adolescents: A systematic review. Clin Psychol Rev. 2025;119:102586.

104. Mastrogiuseppe E, Alisi L, Romaniello A, Caterino L, Spagnoli A, Marenco M, et al. Managing Cutibacterium acnes endophthalmitis after cataract surgery: A systematic review and meta-analysis. Surv Ophthalmol. 2025.

105. Mediouni S, Ndione C, Parmley EJ, Poder TG, Carabin H, Aenishaenslin C. Systematic review on evaluation tools applicable to One Health surveillance systems: A call for adapted methodology. One Health. 2025;20:100995.

106. Merida DM, Acosta-Reyes J, Bayan-Bravo A, Moreno-Franco B, Laclaustra M, Guallar-Castillon P. Phthalate exposure and subclinical carotid atherosclerosis: A systematic review and meta-analysis. Environ Pollut. 2024;350:124044.

107. Mohamad Tawpik NHN, Ahmad Mizher HA, Zaini S. Mobile Application Intervention Effectiveness in Improving Hypertensive Patients Medication Adherence: A Systematic Review. Journal of Pharmacy. 2025;5(1):115-31.

108. Mohammadi MM, Abdollahzadeh N. The effect of chamomile on postoperative pain: A systematic review and meta-analysis. Heliyon. 2025;11(7).

109. Mohammed SP, Deepika J, Sritharan N, Ravichandran V, Prasanthrajan M, Kannan P. A systematic literature review on artificial intelligence in transforming precision agriculture for sustainable farming: Current status and future directions. Plant Science Today. 2025.

110. Morais Fd, Goldoni D, Kautzmann TR, Jaques PA. Sensor-free Affect Detection in Learning Environments: A Systematic Literature Review. Revista Brasileira de Informática na Educação. 2024;32:679-717.

111. Motalebi A, Khondoker MAH, Kabir G. A systematic review of life cycle assessments of 3D concrete printing. Sustainable Operations and Computers. 2024;5:41-50.

112. Motamedian SR, Ahmadi N, Haeri Boroojeni HS, Jahanbani M, Hartoonian S, Bayati E, et al. The impact of surgical maxillary advancement on speech, breathing and pharyngeal airway dimensions in patients with cleft lip and/or palate: A systematic review and meta-analysis. Journal of Oral and Maxillofacial Surgery, Medicine, and Pathology. 2024;36(6):779-92.

113. Moura BM, Madeira L, Bakker PR, van Harten P, Marcelis M. The association between alterations in motor and cognitive dimensions of schizophrenia-spectrum disorders: A systematic review. Schizophr Res. 2024;267:398-414.

114. Msherghi A, Abuajamieh M, Ekreer M, Alzlitni M, Hajalamin M, Aldieb E, et al. Comparative diagnostic performance of [68 Ga]Ga-FAPI PET/CT and [18 F]FDGPET/CT in biliary tract cancers: a systematic review and meta-analysis. Eur J Nucl Med Mol Imaging. 2025.

115. Muhamad Najemudin MN, Anas Mahmood MZ, Zaini S, Yusof NA. The Effects of Nigella Sativa (Black Seed) in Rhinosinusitis Subjects: A Systematic Review. Journal of Pharmacy. 2025;5(1):132-55.

116. Mumtaz A, Berlas MFT, Malik J, Bhojani MF, Moeed A, Panhwar W, et al. Comparison of Bypass Surgery versus Endovascular Interventions for Peripheral Artery Disease through Systematic Review and Meta-Analysis of Randomized Controlled Trials. J Vasc Interv Radiol. 2025;36(6):933-49 e6.

117. Mundisugih J, Kumar S, Kizana E. Adeno-associated virus-mediated gene therapy for cardiac tachyarrhythmia: A systematic review and meta-analysis. Heart Rhythm. 2024;21(6):939-49.

118. Munoz EG, Parraga-Alava J, Meza J, Proano Morales JJ, Ventura S. Housing fuzzy recommender system: A systematic literature review. Heliyon. 2024;10(5):e26444.

119. Ndongo Sonfack DJ, Tanguay Boivin C, Touzel Deschenes L, Maurand T, Maguemoun C, Berthod F, et al. Bioengineering Human Upper Respiratory Mucosa: A Systematic Review of the State of the Art of Cell Culture Techniques. Bioengineering (Basel). 2024;11(8).

120. Niknejad. A systematic review and meta-analysis comparing the 2019 and 2005 Bosniak classification systems for assessing renal cysts and cystic renal masses: diagnostic accuracy and inter-rater agreement evaluation. 2025.

121. Nobanee H, Vo DTH, Trinh HH, Ullah S. The Big Mac Index: A comprehensive bibliometric and systematic review. Research in Globalization. 2025;10.

122. Novak L, Malinakova K, Trnka R, Mikoska P, Sverak T, Kiiski H, et al. Neural bases of social deficits in ADHD: A systematic review. Does the Theory of Mind matter? Brain Res Bull. 2024;215:111011.

123. Nugroho AN, Soetrisno S, Mudigdo A, Yarso KY, Indarto D, Wahyudi AZ, et al. Innovative strategies in bile duct repair: Assessing efficacy and safety across varied graft techniques - A systematic review. Surg Open Sci. 2025;24:5-15.

124. Okenwa-Vincent EE, Riel N, Pagani LS. A systematic review of risks associated with environmental change on refractive and non-refractive ocular health: Special focus on Africa. Hygiene and Environmental Health Advances. 2025;14.

125. Oliveira VH, Diez S, Dolbeth M, Coelho JP. Restoration of degraded estuarine and marine ecosystems: A systematic review of rehabilitation methods in Europe. J Hazard Mater. 2024;469:133863.

126. Olsen MN, Halse AK, Skeie E, Lein RK, Nilsen RM, Tangvik RJ. Effect of dietary interventions on nutritional status in patients with rheumatoid arthritis and spondyloarthritis - A systematic review and meta-analysis. Clin Nutr. 2024;43(4):926-35.

127. Oudmaijer CAJ, Komninos DSJ, Hoeijmakers JHJ, JNM IJ, Vermeij WP. Clinical implications of nutritional interventions reducing calories, a systematic scoping review. Clin Nutr ESPEN. 2024;63:427-39.

128. Palmer V. Systematic literature review on insider threat: Is the Australian aviation industry complacent or just not understanding insider threat? Journal of the Air Transport Research Society. 2025;4.

129. Parappallil Mathew B, Bangwal D. People centric governance model for smart cities development: A systematic review, thematic analysis, and findings. Research in Globalization. 2024;9.

130. Parenteau AM, Hang S, Swartz JR, Wexler AS, Hostinar CE. Clearing the air: A systematic review of studies on air pollution and childhood brain outcomes to mobilize policy change. Dev Cogn Neurosci. 2024;69:101436.

131. Patil S, Pflugradt N, Weinand JM, Stolten D, Kropp J. A systematic review of spatial disaggregation methods for climate action planning. Energy and AI. 2024;17.

132. Pettemeridou E, Loizidou M, Trajkovic J, Constantinou M, De Smet S, Baeken C, et al. Cognitive and Psychological Symptoms in Post-COVID-19 Condition: A Systematic Review of Structural and Functional Neuroimaging, Neurophysiology, and Intervention Studies. Archives of Rehabilitation Research and Clinical Translation. 2025.

133. Pezzoli P, Therond A, Nikolic M, Watts SK, Guimond S, Seto MC. Reducing antisocial behavior through cognitive training: A systematic review and meta-analysis. Aggression and Violent Behavior. 2024;79.

134. Pino MD, Rivero P, Taylor A, Gabriel R. Impact of depression and cardiovascular risk factors on cognitive impairment in patients with atrial fibrillation: A Systematic review and meta-analysis. Arch Gerontol Geriatr. 2025;128:105601.

135. Pinte L, Dima A, Draghici A, Caraghiulea M, Zamfir-Gradinaru IA, Baicus C. Autoimmunity, a relevant exclusion criterion in the development of mRNA-based compounds: A systematic review of clinical trials registries. Autoimmun Rev. 2024;23(12):103670.

136. Poncio FP. Navigating techniques in job recommender systems on internship profile matching: a systematic review. Journal of Research in Innovative Teaching & Learning. 2024;17(2):352-67.

137. Poursalehian M, Hassanzadeh A, Lotfi M, Mortazavi SMJ. Conversion of a Failed Hip Hemiarthroplasty to Total Hip Arthroplasty: A Systematic Review and Meta-Analysis. Arthroplast Today. 2024;28:101459.

138. Pravednikov A, Perkovic S, Lagerkvist CJ. Main factors influencing the perceived health risk of endocrine-disrupting chemicals: A systematic literature review. Environ Res. 2024;262(Pt 1):119836.

139. Qaderi K, Shamsabadi A, Haseli A, Ghane Ezabadi S, Asadi L, Jesmani Y, et al. Changes in screening, diagnosis, management, and outcomes of gestational diabetes during the COVID-19 pandemic: A systematic review. Heliyon. 2024;10(11):e31943.

140. Quintar NA, Escribano JG, Manrique GM. How technology augments Dance Movement Therapy for Autism Spectrum Disorder: A systematic review for 2017–2022. Entertainment Computing. 2025;52.

141. Rahman H, Ghosh P, Nasir F, Khan MA, Rehman N, Sharma S, et al. Short- and intermediate-term outcomes of transcatheter aortic valve replacement in low-risk patients: A meta-analysis and systematic review. Int J Cardiol Heart Vasc. 2024;53:101458.

142. Rezaeizadeh G, Mansournia MA, Keshtkar A, Farahani Z, Zarepour F, Sharafkhah M, et al. Maternal education and its influence on child growth and nutritional status during the first two years of life: a systematic review and meta-analysis. EClinicalMedicine. 2024;71:102574.

143. Rijcken E, Zervanou K, Mosteiro P, Scheepers F, Spruit M, Kaymak U. Machine learning vs. rule-based methods for document classification of electronic health records within mental health care—A systematic literature review. Natural Language Processing Journal. 2025;10.

144. Rohayati T, Dwidienawati D, Aniesta D. Systematic Literature Review of Antecedents and Outcomes of Employee Experience. Wseas Transactions on Computer Research. 2024;13:14-26.

145. Roman MP, Ciortea R, Doumouchtsis SK, Din R, Malutan AM, Bucuri CE, et al. A quality assessment and systematic review of clinical practice guidelines on hormone replacement therapy for menopause using the AGREE II instrument. Eur J Obstet Gynecol Reprod Biol. 2024;303:294-301.

146. Sáenz-Leandro R, Fernández-Ardèvol M. The politics of policy change in platform capitalism: A systematic review of the literature on the regulation of Transportation Network Companies (TNCs). Platforms & Society. 2024;1.

147. Sahraian MA, Emami S, Ataei S, Ghalandari N. Exploring autoimmune endocrine diseases induced by monoclonal antibodies used as multiple sclerosis pharmacotherapy: a systematic review. Naunyn Schmiedebergs Arch Pharmacol. 2025;398(2):1111-28.

148. Senequier A, Draper-Rodi J, Alvarez Bustins G, Braithwaite FA, Brown J, Corcoran D, et al. Investigating the trustworthiness of randomized controlled trials in osteopathic research: a systematic review with meta-analysis. J Clin Epidemiol. 2025;183:111788.

149. Shalabi D, Plume M, Berggren H, Nguyen H, Steingrimsson S, Larson T, et al. Effects of group psychotherapy compared to waiting list or other active intervention on depression and other clinical outcomes in adults with autism spectrum disorder: A systematic review. Journal of Affective Disorders Reports. 2025;21.

150. Siewers K, Svaerke K, Rosenørn AE, Christensen H. Nutritional care in rehabilitation and acute care of stroke patients: a systematic review of clinical practice guidelines. Frontiers in Stroke. 2025;4.

151. Simaey M, De Decker I, Vanlauwe F, Blondeel P, Monstrey S, Claes KEY. The added value of cultured cells in burn treatment: A systematic review. Burns. 2024;50(9):107247.

152. Sjobom U, Hellqvist T, Humayun J, Nilsson AK, Gyllensten H, Hellstrom A, et al. Circulating VEGF-A Levels in Relation to Retinopathy of Prematurity and Treatment Effects: A Systematic Review and Meta-Analysis. Ophthalmol Sci. 2024;4(6):100548.

153. Steller RS, Recklies EA, Schweizer-Ries P. Shaping transformation: Discourse analysis and systematic review of socio-psychological factors in hydrogen technology acceptance. International Journal of Hydrogen Energy. 2024;81:1421-41.

154. Suwannasom N, Sriaksorn N, Thepmalee C, Thephinlap C, Tanamatayarat P, Khoothiam K, et al. Efficacy and safety of nanoparticle albumin-bound paclitaxel in advanced non-small cell lung cancer: A systematic review and meta-analysis of clinical trials and observational studies. Heliyon. 2023;9(11):e21903.

155. Talha M, Nejadhashemi AP, Moller K. Soft computing paradigm for climate change adaptation and mitigation in Iran, Pakistan, and Turkey: A systematic review. Heliyon. 2025;11(2):e41974.

156. Taliento C, Scutiero G, Arcieri M, Pellecchia G, Tius V, Bogani G, et al. Simple hysterectomy versus radical hysterectomy in early-stage cervical cancer: A systematic review and meta-analysis. Eur J Surg Oncol. 2024;50(4):108252.

157. Taveekitworachai P, Chanmas G, Paliyawan P, Thawonmas R, Nukoolkit C, Dajpratham P, et al. A systematic review of major evaluation metrics for simulator-based automatic assessment of driving after stroke. Heliyon. 2024;10(12):e32930.

158. Teufer B, Grabner-Kräuter S, Bachner C. Sustainable development outcomes of alternative consumer networks – A systematic review and logic model development. Cleaner and Responsible Consumption. 2024;15.

159. Torghabeh FD, Javadi B, Sahebkar A. Dietary anethole: a systematic review of its protective effects against metabolic syndrome. J Diabetes Metab Disord. 2024;23(1):619-31.

160. Tran HM, Tsai FJ, Lee KY, Wang YH, Yang FM, Ho SC, et al. Extreme temperature increases the risk of COPD morbimortality: A systematic review and meta-analysis. Sci Total Environ. 2025;958:178087.

161. Umans E, Boogaerts M, Vergauwe B, Verest A, Van Calenbergh S. Vaginal foreign body in the pediatric patient: A systematic review. Eur J Obstet Gynecol Reprod Biol. 2024;297:153-8.

162. Utama DM, Umamy SZ, Al-Imron CN. No-Wait Flow Shop scheduling problem: a systematic literature review and bibliometric analysis. RAIRO - Operations Research. 2024;58(2):1281-313.

163. Valizadeh P, Jannatdoust P, Pahlevan-Fallahy MT, Hassankhani A, Amoukhteh M, Bagherieh S, et al. Diagnostic accuracy of radiomics and artificial intelligence models in diagnosing lymph node metastasis in head and neck cancers: a systematic review and meta-analysis. Neuroradiology. 2025;67(2):449-67.

164. van Amsterdam J, van den Brink W. Sweet-liking and sugar supplementation as innovative components in substance use disorder treatment: A systematic review. J Psychopharmacol. 2025;39(4):328-38.

165. van Eickels RL, Siegel M, Juhasz AJ, Zemp M. The parent-child relationship and child shame and guilt: A meta-analytic systematic review. Child Dev. 2025;96(3):907-29.

166. Vargas R, Lizano-Barrantes C, Romero M, Valencia-Clua K, Narvaez-Narvaez DA, Sune-Negre JM, et al. The piper at the gates of brain: A systematic review of surface modification strategies on lipid nanoparticles to overcome the Blood-Brain-Barrier. Int J Pharm. 2024;665:124686.

167. Veerman E, Danbury E, Duarte JdS, Volman M, Gaikhorst L. A systematic review of empirical studies into multilingual pedagogies and their outcomes in primary education. Journal of Multilingual and Multicultural Development. 2025:1-26.

168. Villarreal-Zegarra D, Yllescas-Panta T, Malaquias-Obregon S, Damaso-Roman A, Mayo-Puchoc N. Effectiveness of animal-assisted therapy and pet-robot interventions in reducing depressive symptoms among older adults: A systematic review and meta-analysis. Complement Ther Med. 2024;80:103023.

169. Virtanen M, Kerimaa H, Mannikko N, Mannisto M, Paalimaki-Paakki K, Lahtinen M, et al. Digital components and interaction types in counseling interventions for childhood and adolescent obesity: A systematic review. Int J Nurs Sci. 2025;12(2):123-9.

170. Vo TMH, Huynh TPL, Tamas P, Woillez M-N, Espagne E, Umans L, et al. How consistent are adaptation strategies with ongoing climatic and environmental changes in the Vietnamese Mekong Delta: A systematic review. Environmental Science & Policy. 2025;168.

171. Vorderobermeier A, Abel J, Sailer M. Theoretical foundations and approaches in research on educational escape rooms: A systematic review. Educational Research Review. 2024;44.

172. Wang B, Shi H, Yao Y, Liu S, Shi C. The Association Between Renal Desaturation Measured Using Near-Infrared Spectroscopy and Postoperative Acute Kidney Injury: A Systematic Review. J Cardiothorac Vasc Anesth. 2024;38(11):2606-12.

173. Wang JY, Chen Y, Dong R, Li S, Peng JM, Hu XY, et al. Extracorporeal vs. conventional CPR for out-of-hospital cardiac arrest: A systematic review and meta-analysis. Am J Emerg Med. 2024;80:185-93.

174. Wang Y, Yang J, Hu X, Shi J, Deng J. Different types of pesticide exposure and lung cancer incidence in the Agricultural Health Study cohort: A systematic review and meta-analysis. Arch Environ Occup Health. 2024;79(7-8):263-72.

175. Woldan-Gradalska P, Gradalski W, Gunnarsson RK, Sundvall PD, Rystedt K. Is Streptococcus pyogenes a pathogen or passenger in uncomplicated acute sore throat? A systematic review and meta-analysis. Int J Infect Dis. 2024;145:107100.

176. Wongsa C, Wongyikul P, Chokevittaya P, Nititammaluk A, Soe KK, Phinyo P, et al. Subtype prevalence and treatment implication in adolescents and adults with mild-to-moderate asthma: Systematic review and meta-analysis. J Allergy Clin Immunol Glob. 2025;4(1):100366.

177. Wu T, Yan J, Nie K, Chen Y, Wu Y, Wang S, et al. Microfluidic chips in female reproduction: a systematic review of status, advances, and challenges. Theranostics. 2024;14(11):4352-74.

178. Xu Z, Feng Z, Babaeian Jelodar M, Guo BHW. Augmented reality applications in construction productivity: A systematic literature review. Advanced Engineering Informatics. 2024;62.

179. Yaghoobpoor S, Fathi M, Ghorani H, Valizadeh P, Jannatdoust P, Tavasol A, et al. Machine learning approaches in the prediction of positive axillary lymph nodes post neoadjuvant chemotherapy using MRI, CT, or ultrasound: A systematic review. Eur J Radiol Open. 2024;12:100561.

180. Yanan L, Ismail MA, Aminuddin A. How has rural tourism influenced the sustainable development of traditional villages? A systematic literature review. Heliyon. 2024;10(4):e25627.

181. Ye G, Ying Y, Shen B, Liu J, Lu J. Effect of intraoral photobiomodulation therapy on pain perception associated with local anaesthesia infiltration: a systematic review and meta-analysis of randomized controlled trials. Int J Oral Maxillofac Surg. 2025;54(1):82-92.

182. Yslado Mendez RM, Sanchez-Broncano J, Mendoza Ramirez GD, Villarreal-Zegarra D. Prevalence and factors associated with burnout syndrome in Peruvian health professionals before the COVID-19 pandemic: A systematic review. Heliyon. 2024;10(9):e30125.

183. Yu H, Li M, Qian G, Yue S, Ossowski Z, Szumilewicz A. A Systematic Review and Bayesian Network Meta-Analysis Comparing In-Person, Remote, and Blended Interventions in Physical Activity, Diet, Education, and Behavioral Modification on Gestational Weight Gain among Overweight or Obese Pregnant Individuals. Adv Nutr. 2024;15(7):100253.

184. Zaffanello M, Pietrobelli A, Nosetti L, Antoniazzi F, Frassoldati R, Piacentini G. Intranasal Corticosteroids and Oral Montelukast for Paediatric Obstructive Sleep Apnoea: A Systematic Review. Pharmaceutics. 2025;17(5).

185. Zaka A, Mustafiz C, Mutahar D, Sinhal S, Gorcilov J, Muston B, et al. Machine-learning versus traditional methods for prediction of all-cause mortality after transcatheter aortic valve implantation: a systematic review and meta-analysis. Open Heart. 2025;12(1).

186. Zebrowitz E, Dadoo S, Brabant P, Uddin A, Aifuwa E, Maraia D, et al. The impact of artificial intelligence on large vessel occlusion stroke detection and management: A systematic review meta-analysis. Intelligence-Based Medicine. 2024;10.

187. Zhao J, Peng Y, Lin Z, Gong Y. Association between Mediterranean diet adherence and Parkinson's disease: a systematic review and meta-analysis. J Nutr Health Aging. 2025;29(2):100451.

188. Zhao Q, Deng Y, Gong R, Chen T, Yang L. Association between dietary inflammatory index and risk of fatty liver disease: A systematic review and meta-analysis. Dig Liver Dis. 2024;56(4):541-50.
